# Prepregs for Temperature Resistant Composites

**DOI:** 10.3390/ma12234012

**Published:** 2019-12-03

**Authors:** Eliška Haincová, Pavlína Hájková, Jan Kohout

**Affiliations:** Unipetrol Centre for Research and Education, Revoluční 84, 40001 Ústí nad Labem, Czech Republic; Pavlina.Hajkova@unicre.cz (P.H.); Jan.Kohout@unicre.cz (J.K.)

**Keywords:** composite, carbon fiber, aluminosilicate matrix, prepreg, temperature resistant, tensile strength

## Abstract

In this paper, carbon fabric reinforced inorganic matrix composites were prepared. The inorganic matrix based on alkali activated aluminosilicate was used because of its resistance to fire and the temperatures up to 1000 °C. Influence of heat treatment of fabric, high temperature treatment of composite and preparation method on the mechanical properties and morphology of the composites were studied. The preparation of composites with the subsequent steps of impregnation, layering and curing of the composites was compared with the prepreg preparation method, which separates the impregnation of the reinforcement from the production of the composite. The SEM photographs show no differences in morphology between composites prepared from heat treated fabric and composites prepared from original fabrics. All four series of samples were comparatively saturated with matrix. Despite this, tensile properties of heat-treated fabric composites were negatively affected. While composites with heat-treated fabric reached the tensile strength up to 274 MPa, composites prepared without heat-treated fabric exhibited strengths higher than 336 MPa. Samples exposed to temperatures reaching 600 °C retained up to 40% of their original strength. The effect of composite preparation method on the tensile properties of the composites has not been proved.

## 1. Introduction

Fiber-reinforced polymer composites are widely used as lightweight structural materials in aerospace, naval, navigation, electronics, and automotive industries. In most cases, composites are made from organic matrix and carbon, basalt, or glass fibers. These materials exhibit excellent properties such as high tensile and flexural strength, low density, and corrosion resistance [1,2,3]. The use of nanofiber structures as composite fillers is a recent trend in the field of composites [4,5]. The disadvantage of composites prepared from organic matrix is that they cannot be used at temperatures above 200 °C. This is one of the reasons why an inorganic matrix has been studied in recent years [3]. 

One of the perspective substitutes for an organic matrix for the preparation of composites is inorganic material based on the alkali-activated aluminosilicates (A-matrix), material that can be called geopolymer under certain conditions. A-matrix is formed by mixing powdered aluminosilicates with a liquid alkaline activator. A liquid alkali silicate and alkali metal hydroxide solution are usually used to dissolve material containing Si and Al such as metakaolin [6,7,8,9]. A-matrix is often reinforced by solid materials to improve its properties [9]. The properties of prepared A-matrix depend on the type and amount of aluminosilicate, activator, additive, water, Si/Al molar ratio, Na/Al molar ratio, Na^+^, or K^+^ content and the curing conditions [9,10,11,12]. A-matrix can be cured at the low temperatures (even at a room temperature). This material has good mechanical properties and it is resistant to chemicals and temperatures up to 1000 °C.

Various kinds of composites with inorganic matrix based on alkali-activated aluminosilicates, including particulate [13,14], continuous fiber [15,16] and short fiber [6,17], were investigated in many studies. Glass [18,19], carbon [15,16,19] and basalt [17,19,20] fabrics or unidirectional continuous fibers were usually layered in 6–16 layers [15,16]. Mechanical properties were studied on samples cured at laboratory temperature and even after high-temperature heat treatment [6,14,21]. Commonly, compressive strength [14,17,21], flexural strength [6,14,15,16,19], Young’s modulus [6,15,19], tensile properties [22,23], and impact resistance [18,19] were investigated on composites with A-matrix. The properties of A-matrix composites depended on the type of fiber reinforcement, number of layers of fiber reinforcement, composition of A-matrix, A-matrix/fiber reinforcement weight ratio, and cure conditions of composites. Krystek et al. [23] prepared composites with A-matrix and carbon fabric. The prepared samples reached a tensile strength up to 265 MPa.

Epoxy resins are usually used for carbon fiber sizing. Unfortunately, the carbon fibre sizing has made it difficult to apply the A-matrix to the surface of the fiber reinforcement; therefore, many authors removed it in various ways. Yan et al. [24] washed the carbon fibers by an ultrasonic vibrator in acetone and then dried them in oven at 60 °C for 5 h. Lin et al. [25] separated the short carbon fibers by an ultrasonic vibrator in ethanol, then the fibers were filtered out by a wire sieve to get sheet-like short carbon fiber preforms with a thickness in the range of 0.15–0.2 mm. Yuan et al. [26] treated the fibers at 370 °C in air atmosphere for 2 h.

Composites can be also made from pre-impregnated fabric reinforcements called prepregs. In that case, the fabrics are commonly laminated by resin and stored for several months in a tempered equipment before layering. This method is typical for composites with organic matrix [18,27]. The advantages of composites prepared from prepregs are easy manipulation with fiber reinforcement, better fiber reinforcement saturation with epoxy matrix, and the possibility of dividing the preparation of composites for fiber reinforcement impregnation and layering of pre-impregnated reinforcement. The preparation of geopolymer prepregs is not yet widely published.

This work is focused on a comparison of mechanical properties of geopolymer composite materials prepared in two ways. Conventional lamination of the carbon fabric, which is subsequently layered into a composite, is compared to the production of prepreg composites. The preparation of prepregs is part of this work. The properties of geopolymer composites made from untreated carbon fabrics and treated carbon fabric at 300 °C were also compared. The tensile strength, modulus of elasticity and the composite structure were studied on geopolymer composite samples after thermal exposure at a temperature up to 800 °C.

## 2. Materials and Methods

Carbon plain weave fabric with the area weights of 200 g/m^2^ (Carbon fabric eSpread 200 CHT, Porcher Industries, La Voulte-sur-Rhône, France) was used as the reinforcement for composites. Commercial metakaolinite-rich material produced by the calcination of kaolinitic claystone in rotary kiln at c. 750 °C (České lupkové závody, a.s., Nové Strašecí, Czech Republic) [28], silica fume (České lupkové závody, a.s., Nové Strašecí, Czech Republic), commercial potassium water glass with molar ratio SiO_2_:M_2_O equal to 1.7 (Vodní sklo, a.s., Prague, Czech Republic), potassium hydroxide flakes (Lach-Ner, s.r.o., Neratovice, Czech Republic), boric acid (Penta, s.r.o., Prague, Czech Republic), and distilled water were used for preparation of A-matrix.

**Figure 1:**
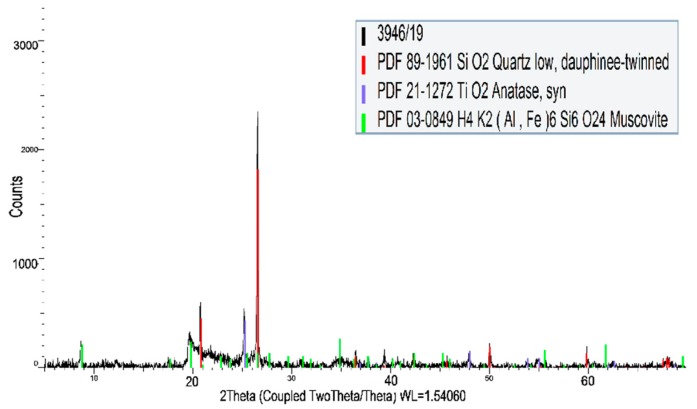
X-ray powder diffraction (XRD) analysis of metakaolinite-rich material.

**Figure 2:**
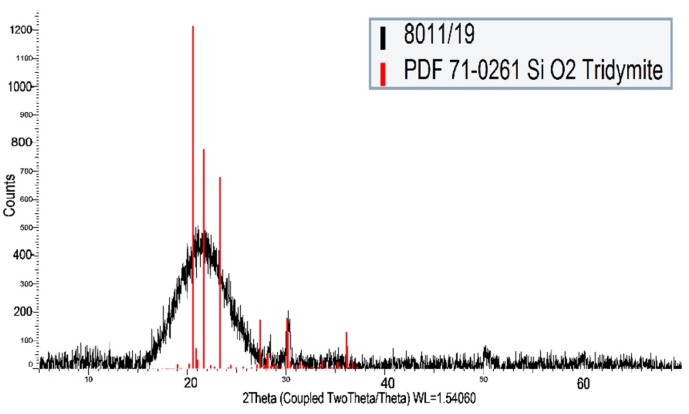
XRD analysis of silica fume.

The chemical compositions of powdered raw materials determined by X-ray fluorescence (XRF, Bruker S8 Tiger, Billerica, MA, USA) can be seen in Table 1. The structural properties shown in Figure 1 and Figure 2 were specified by a BRUKER D8 Advanced X-ray diffraction system (XRD) equipped with a BRUKER SSD 160 detector and operating with Cu-Kα radiation. Size distribution of powdered raw materials was determined by a Mastersizer 2000 laser diffraction particle size analyser (MALVERN Instruments, Malvern, United Kingdom). The morphology of carbon fabric and prepared composites was observed by a scanning electron microscope (SEM, JEOL JSM-IT500HR, Tokyo, Japan). The conventional acid-base titration method and an inductively coupled plasma optical emission spectrometer OPTIMA 8000 (PerkinElmer, Waltham, MA, USA) were used for chemical analysis of water glass (Table 1). 

Alkaline activator was prepared with composition of molar ratio SiO_2_/K_2_O = 1.15 and K_2_O/B_2_O_3_ = 5.15 by mixing the commercial potassium water glass, potassium hydroxide solution (KOH:H_2_O = 1:1 in weight ratio), solid boric acid, and distilled water. Activator was mixed in a blender (Kenwood KVL8400S Chef XL Titanium, Havant, United Kingdom) for 24 h and stored in the fridge at 5 °C for two days. Then, the metakaolinite-rich material and silica fume were added to the alkaline activator. This mixture of A- matrix with composition of SiO_2_/Al_2_O_3_ = 33.9, K_2_O/Al_2_O_3_ = 3.98, H_2_O/K_2_O = 12.1 (molar ratio) was blended c. 30 min, stored in a freezer at −18 °C for 24 h and then used to prepare four six-layer composite plates.

**Figure 3:**
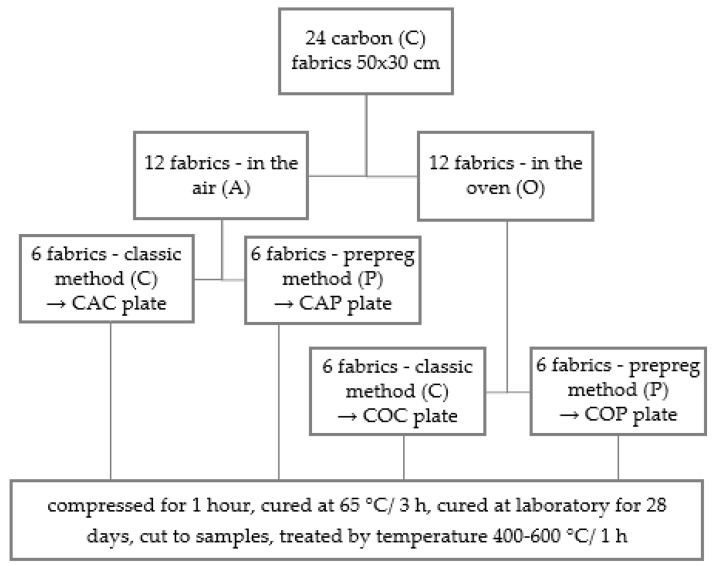
Scheme of composite plates preparation.

Four six-layer composite plates were prepared from 24 pieces of 50 cm × 30 cm carbon fabric. Composite series were different in the combination of fabric pre-treatment and preparation method. For clarity, the scheme of composites preparation with the sample marking system is shown in Figure 3. Twelve source pieces of carbon fabric for preparation of the first and second composite plates were kept in the air (CA) until the composite preparation. The remaining twelve pieces of fabric were placed into an oven at 300 °C for one hour to remove the epoxy layer from the surface of the carbon fibers (CO). Then, all fabrics were impregnated in a conventional manner with the A-matrix using a paint roller. Six impregnated CA fabric pieces were used for composite plate by classic method (CAC). The CAC plate was prepared by stacking impregnated fabrics one by one. The other six pieces of fabric were used for preparation of composite plate by prepreg method (CAP). In this case, the impregnated carbon fabrics were individually placed between two pieces of plastic foil (two pieces for every fabric) to prepare the prepregs. Prepregs were stored in a freezer at −18 °C. After seven days, the prepregs were taken out of the freezer, stripped of plastic foil, and used for preparation of composites by stacking one by one to get the CAP composite plate as in the case of CAC plate. Twelve impregnated carbon CO fabrics were used for preparation of the COC plate by a classic method and the COP plate by the prepreg method in the same way as CAC and CAP composite plates. Every prepared composite plate was placed between two pieces of peel-ply fabric, wrapped in a plastic foil, compressed at 440 kPa for one hour and then cured in the oven at 65 °C for 3 h. After this time, the plates were unwrapped from the plastic foil and peel-ply fabric and finally cured for 28 days at laboratory conditions. The fabric mass fraction of the plates is presented in Table 2.

**Figure 4:**
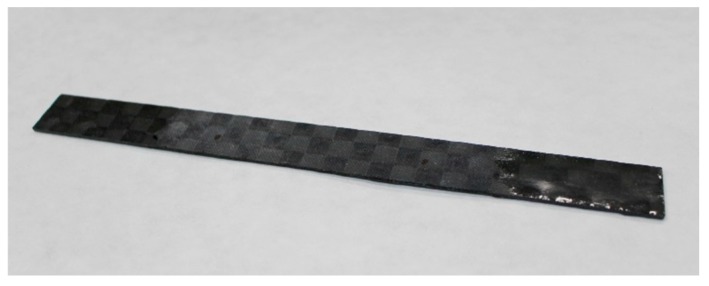
Composite sample.

**Figure 5:**
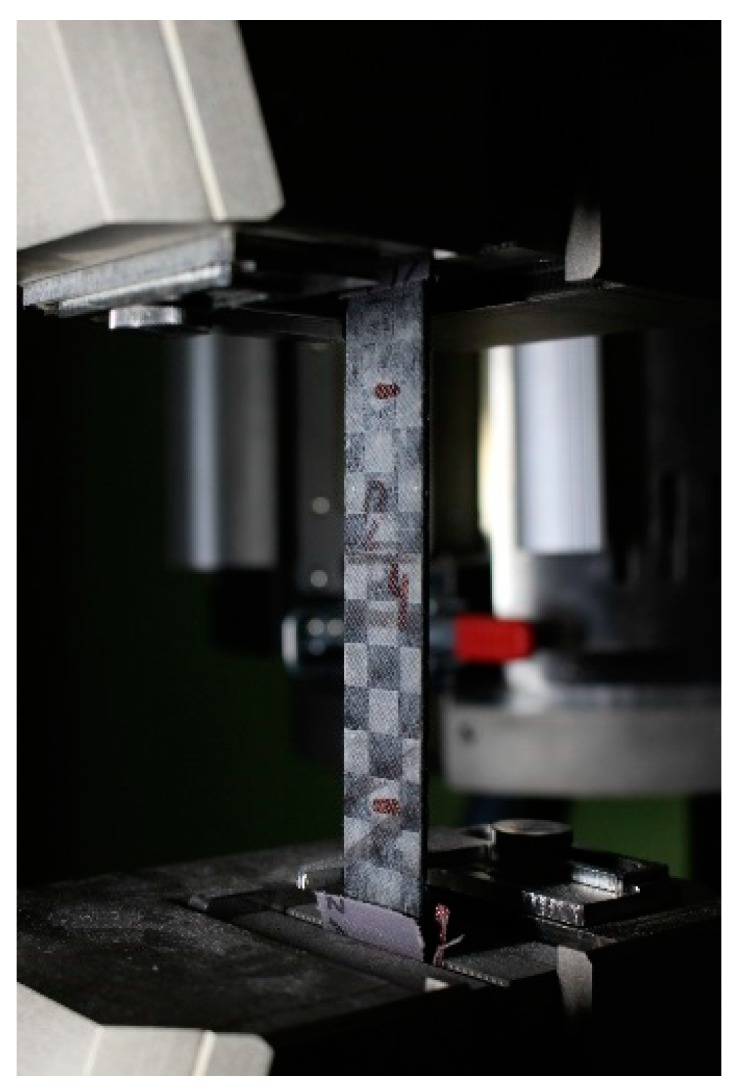
Measurement of tensile properties of the composite sample using the LabTest 6.200.

Four prepared composite plates were cut into 250 × 25 mm samples (Figure 4) by water jets. The obtained samples were kept at a laboratory temperature (LT) or treated with temperatures of 400, 500, and 600 °C for one hour. The treated temperatures were added to the sample names (CAC-LT, COP-400, etc.). All samples were tested for tensile strength and modulus of elasticity using the universal testing machine LabTest 6.200 (maximum load of the sensor 200 kN) at a loading speed of 2 mm/min. (LaborTech, s.r.o., Opava, Czech Republic) complying with ASTM 3039 (Figure 5). The ends of the samples were reinforced with epoxy resin coating and covered with sandpaper to protect the composite surface from sharp grips. Prepared composite samples were studied by a scanning electron microscope. 

## 3. Results and Discussion

### 3.1. A-Matrix

**Figure 6:**
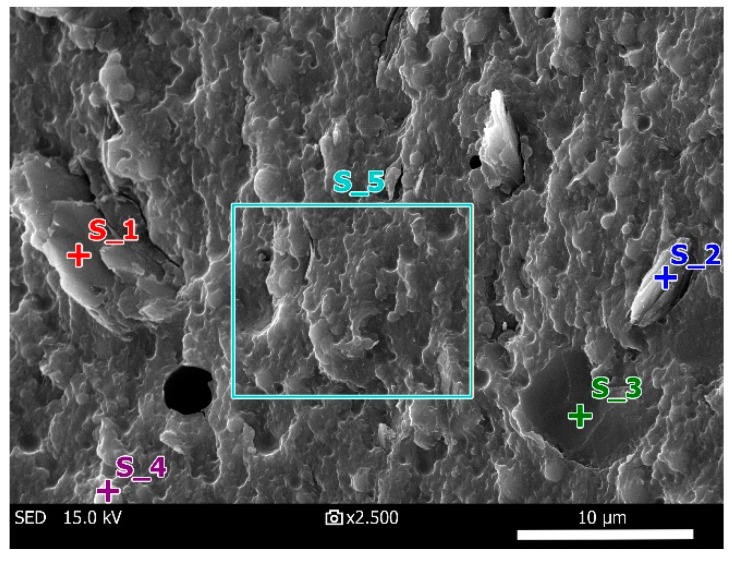
SEM photograph of the A-matrix.

**Figure 7:**
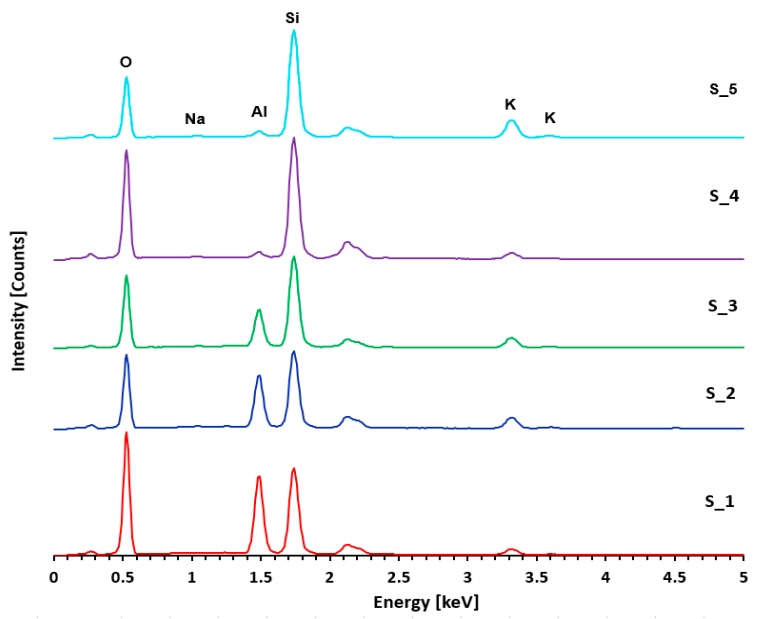
Spectrum of A-Matrix (Figure 6) obtained by EDS.

Figure 6 obtained by SEM presents morphology of the A-matrix. Four points and one bounded area, where chemical analysis was performed, are shown in the figure. The chemical composition results can be seen in Figure 7 and the exact values are reported in Table 3. High content of Al_2_O_3_ and SiO_2_ with lower content of K_2_O and Na_2_O (points S_1, S_2, S_3) indicate presence of metakaolin, and the increased SiO_2_ content (S_4) induces undissolved SiO_2_ particle, probably covered by a thin layer of dissolved components. The bounded area (S_5) contains all components of the A-matrix in a proportion corresponding to the amount of material preparation.

### 3.2. Carbon Fabric

**Figure 8:**
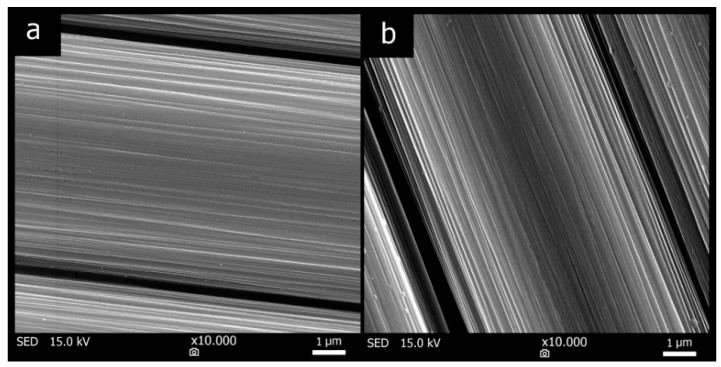
SEM of fiber surface of fibers (**a**) without heat treatment and (**b**) after 300 °C/1 h.

The carbon fiber surface studied by SEM is presented in Figure 8. Figure 8a shows the surface of the fibers not treated with elevated temperature, and surface of the fibers treated at 300 °C for one hour is showed in Figure 8b. The SEM photographs indicate that the differences in surface between these fibers are minimal and that heat treatment of the carbon fabric probably did not adversely affect the surface. Both types of fibers were subjected to the tensile strength test to compare the tensile properties. The obtained values (Table 4) confirmed that the exposure to temperature of 300 °C did not lead up to the deterioration of fibers’ tensile properties.

### 3.3. Composite Surface

**Figure 9:**
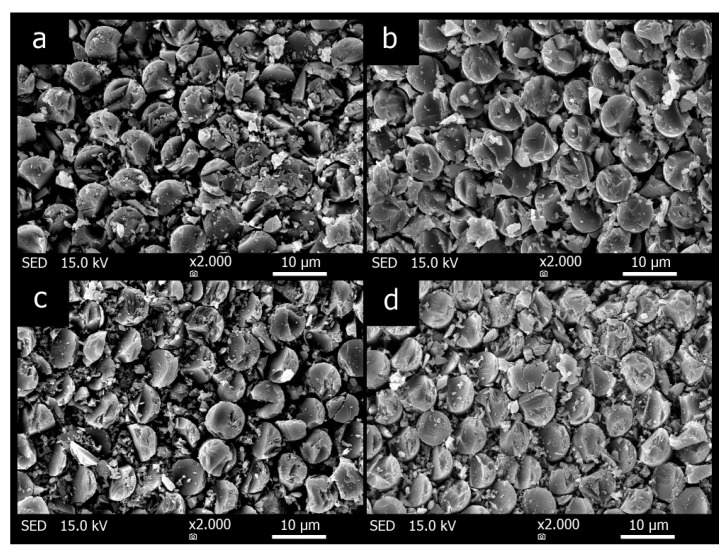
Cross-section micrographs of the composite plates (**a**) CAC, (**b**) COC, (**c**) CAP, (**d**) COP.

Figure 9 presents the morphology of prepared composites. These SEM photos of the sample cross sections confirm that the carbon fiber distribution is similar in all composite samples, and the inorganic matrix surrounds the individual fibers of the fabrics independently of the thermal treatment of the fabrics or the preparation method. The uniform fiber saturation is one of the reasons for the high strength of composites. 

**Figure 10:**
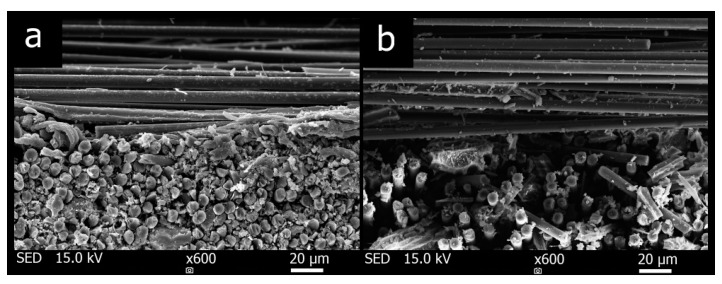
Cross-section micrographs of the composite plates (**a**) COC-LT and (**b**) COC-500.

The fiber distribution of the fabric in the prepared composite is shown in the Figure 10. The comparison of the composite without heat treatment COC-LT (**a**) and after the heat treatment COC-500 (**b**) can be seen here. The embrittlement of the whole sample matrix is more significant with increasing the temperature, the fiber-bonding matrix cracking, crumbling and moving away from the fiber surface. This leads to a decrease in the tensile strength of the composites.

### 3.4. Tensile Properties of Composites

**Figure 11:**
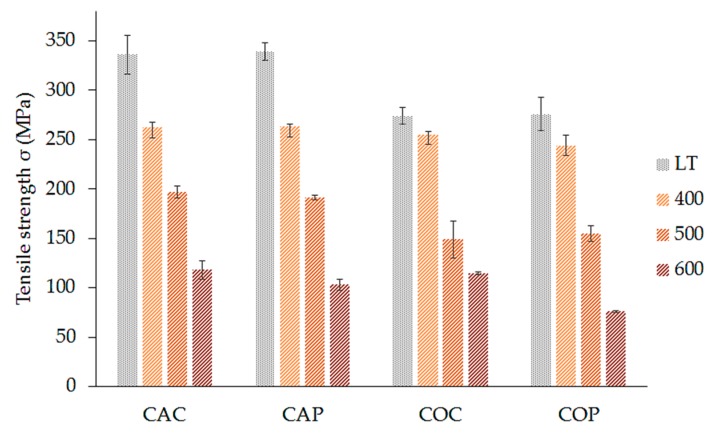
Influence of fiber treatment, preparation method, and temperature treatment on the tensile strength of the composite plates.

**Figure 12:**
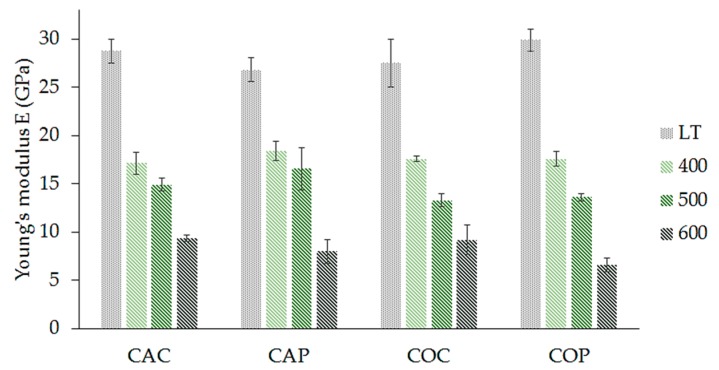
Influence of fiber treatment, preparation method, and temperature treatment on the Young’s modulus of the composite plates.

The influence of the composites’ preparation method on their tensile properties was studied on four series of samples CAC, CAP, COC, and CAP composite plates. Changes in tensile properties due to heat treatment of the fabrics and use of two methods of plate preparation and finally influence of high temperature treatment on composite samples were observed. Tensile strength, strain, and Young’s modulus were recorded for temperatures ranging from laboratory temperature to 600 °C on 30-day-old samples. The average values of tensile properties of composites are summarized in Figure 11 and Figure 12.

As expected [21,22,23], the composite samples cured at laboratory temperature in all four series (CAC-LT, CAP-LT, COC-LT, COP-LT) had the highest tensile strength and the strength decreased with increasing cure temperature. In Figure 11, we can see a significant difference in tensile strength (up to 63 MPa) between composites made of heat-treated fabrics (COC-LT, COP-LT) and composites made from fabrics with no heat treatment (CAC-LT, CAP-LT). The decrease in tensile strength could be due to the interaction of the alkaline matrix with the temperature exposed fabrics. This was visible only for samples cured at laboratory temperature. In case of the samples cured at temperatures 400–600 °C, the differences diminished. Each of these composite samples was treated by high temperature, so damages were similar. The above described facts will be examined in the following study. The measured values correlate with Krystek et al. [23].

**Figure 13:**
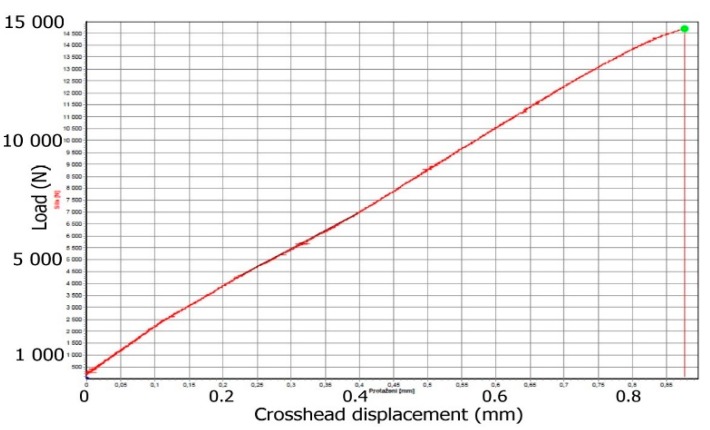
Typical load vs. crosshead displacement profile of prepared composite.

The COP-600 plate had the lowest tensile strength. Removal of the organic sizing from the fabric in combination with the prepreg method proved to be the composite with the lowest tensile strength. The seven-day long exposure to the alkaline matrix on the temperature treated fibers probably led to the interaction with the fibers and the strength decreased compared to the other plates affected by 600 °C. The typical load vs. crosshead displacement profile of prepared composite is showed in Figure 13. 

The influence of the preparation method on the strength of the composites was not significant. The strength differences were predominantly within the standard deviation. While the classic method is faster, the prepreg method is advantageous in the industrial sphere, where it is necessary to divide production processes into fabric lamination and composite production. The final product can be shaped and layered from the prepreg prepared by the process described in this article to 30 days from the preparation of the matrix.

The behavior of the Young’s modulus is illustrated in Figure 12. Samples cured at laboratory temperature show the highest Young’s modulus. In contrast, samples treated at 600 °C showed the lowest values. In this case, the modulus was c. 1/3 compared to the modulus of samples cured at laboratory temperature. The measured values of four composite series are very similar; the differences are within the standard deviation.

## 4. Conclusions

In this investigation, the carbon fabrics reinforced aluminosilicate matrix composites were prepared by a simple classic method and by the prepreg method. Effects of heat treatment of fabric, high temperature treatment of the composite, and the preparation method on the mechanical properties and morphology of the composites were studied. Results lead to these conclusions:

- All four types of composites showed homogenous microstructure and carbon fabric was well infiltrated by the inorganic aluminosilicate matrix independent of the fiber treatment or preparation method.
- The highest tensile strength was seen in samples prepared without fiber heat treatment, with classic lay-up samples exhibiting a strength of 336 ± 19 MPa and prepreg prepared samples exhibiting a strength of 339 ± 9 MPa.
- The composites lost high tensile strength with increasing curing temperature, but they retained 30–40 % of their original strength at 600 °C.
- Significant decrease in tensile strength of samples with heat treated fabric. Therefore, removal of the organic sizing by elevated temperature did not show any positive effects.
- The method of preparation of composite had no significant effect on the tensile strength or Young’s modulus of the samples. The prepreg method of composite preparation is, in terms of tensile properties, a good substitute for classic composite preparation.

## Figures and Tables

**Table 1:** Composition of powdered raw materials and water glass.

Material	Material Composition (%)								
	H_2_O	SiO_2_	Al_2_O_3_	Na_2_O	K_2_O	CaO	P_2_O_5_	Fe_2_O_3_	ZrO_2_
Metakaolinite-rich material	1.26	52.3	42.6		0.77	0.18	0.08	0.81	
Silica fume	0.62	93.8	0.15		0.04	0.09	0.43		1.56
Potassium water glass	44.5	28.5		1.12	24.2				

**Table 2:** Fabric fraction of the plates.

Plate	Area (m^2^) of 1 Piece of Carbon Fabric (200 g/m^2^)	Weight of Composite Plate (g)	Carbon Fabric Reinforcement (wt. %)
CAC	0.15	536.5	33.6
CAP	0.15	528.5	34.1
COC	0.15	525.0	34.3
COP	0.15	529.6	34.0

**Table 3:** Chemical composition of A-matrix measured by energy-dispersive X-ray spectroscopy (EDS).

Name	Composition (%)				
	Na_2_O	Al_2_O_3_	SiO_2_	K_2_O	Total
S_1	<0.20	37.12	58.10	4.78	100.00
S_2	0.44	29.65	59.06	10.51	100.00
S_3	0.29	21.24	68.83	9.64	100.00
S_4	0.33	3.41	89.75	6.51	100.00
S_5	0.52	3.09	78.08	18.31	100.00

**Table 4:** Tensile properties of carbon fiber.

	Fiber without Heat Treatment	Fiber after 300 °C/1 h
Tensile strength (MPa)	2949.2	3074.1
Standard deviation (MPa)	445.5	322.7

## References

[1] Sun G., Tong S., Chen D., Gong Z., Li Q. (2018). Mechanical properties of hybrid composites reinforced by carbon and basalt fibers. Int. J. Mech. Sci..

[2] Krystek J., Kroupa T., Kottner R. Identification of mechanical properties from tensile and compression tests of unidirectional carbon composite. Proceedings of the 48th International Scientific Conference on Experimental Stress Analysis.

[3] Papakonstantinou C.G., Balaguru P., Lyon R.E. (2001). Comparative study of high temperature composites. Compos. Part B.

[4] Tran T.Q., Lee J.K.Y., Chinnappan A., Jayathilaka W.A.D.M., Ji D., Kumar V.V., Ramakrishna S. (2019). Strong, Lightweight, and Highly Conductive CNT/Au/Cu Wires from Sputtering and Electroplating Methods. J. Mater. Sci. Technol..

[5] Duong H.M., Myint S.M., Tran T.Q., Le D.K. (2020). Post-spinning treatments to carbon nanotube fibers. Carbon Nanotube Fibers and Yarns.

[6] Yan S., He P., Zhang Y., Jia D., Wang J., Duan X., Yang Z., Zhou Y. (2017). Preparation and in-situ high-temperature mechanical properties of Cf-SiCf reinforced geopolymer composites. Ceram. Int..

[7] Silva F.J., Thaumaturgo C. (2003). Fibre reinforcement and fracture response in geopolymeric mortars. Fatique Fract Enging Struct.

[8] Duxson P., Provis J.L., Lukey G.C., Mallicoat S.W., Kriven W.M., van Deventer J.S.J. (2005). Understanding the relationship between geopolymer composition, microstructure and mechanical properties. Colloids Surf. A Physicochem. Eng. Asp..

[9] Kuenzel C., Li L., Vandeperre L., Boccaccini A.R., Cheeseman C.R. (2014). Influence of sand on the mechanical properties of metakaolin geopolymers. Constr. Build. Mater..

[10] Kuenzel C., Vandeperre L.J., Donatello S., Boccaccini A.R., Cheeseman C., Brown P. (2012). Ambient Temperature Drying Shrinkage and Cracking in Metakaolin-Based Geopolymers. J. Am. Ceram. Soc..

[11] Duxson P., Mallicoat S.W., Lukey G.C., Kriven W.M., van Deventer J.S.J. (2007). The effect of alkali and Si/Al ratio on the development of mechanical properties of metakaolin-based geopolymers. Colloids Surf. A Physicochem. Eng. Asp..

[12] Aredes F.G.M., Campos T.M.B., Machado J.P.B., Sakane K.K., Thim G.P., Brunelli D.D. (2015). Effect of cure temperature on the formation of metakaolinite-based geopolymer. Ceram. Int..

[13] Barbosa V.F.F., MacKenzie K.J.D. (2003). Thermal behaviour of inorganic geopolymers and composites derived from sodium polysialete. Mater. Res. Bull..

[14] Kovářík T., Rieger D., Kadlec J., Křenek T., Kullová L., Pola M., Bělský P., Franče P., Říha J. (2017). Thermomechanical properties of particle-reinforced geopolymer composite with various aggregate gradation of fine ceramic filler. Constr. Build. Mater..

[15] He P., Jia D., Lin T., Wang M., Zhou Y. (2010). Effects of high-temperature heat treatment on the mechanical properties of unidirectional carbon fiber reinforced geopolymer composites. Ceram. Int..

[16] He P., Jia D., Wang M., Zhou Y. (2010). Improvement of high-temperature mechanical properties of heat treated Cf/geopolymer composites by Sol-SiO2 impregnation. J. Eur. Ceram. Soc..

[17] Dias D.P., Thaumaturgo C. (2005). Fracture toughness of geopolymeric concretes reinforced with basalt fibers. Cem. Concr. Compos..

[18] Amaro A.M., Pinto M.I.M., Reis P.N.B., Neto M.A., Lopes S.M.R. (2018). Structural integrity of glass/epoxy composites embedded in cement or geopolymer mortars. Compos. Struct..

[19] Samal S., Marvalová B., Petríková I., Vallons K.A.M., Lomov S.V., Rahier H. (2016). Impact and post impact behavior of fabric reinforced geopolymer composite. Constr. Build. Mater..

[20] Kong D.L.Y., Sanjayan J.G. (2008). Damage behavior of geopolymer composites exposed to elevated temperatures. Cem. Concr. Compos..

[21] Bernal S.A., Bejarano J., Garzón C., Mejía de Gutiérrez R., Delvasto S., Rodríguez E.D. (2012). Performance of refractory aluminosilicate particle/fiber-reinforced geopolymer composites. Compos. Part B Eng..

[22] Mills-Brown J., Potter K., Foster S., Batho T. (2013). Thermal and tensile properties of polysialate composites. Ceram. Int..

[23] Krystek J., Laš V., Pompe V., Hájková P. (2018). Tensile and bending test of carbon/epoxy and carbon/geopolymer composites after temperature conditioning. MATEC Web Conf..

[24] Yan S., He P., Jia D., Yang Z., Duan X., Wang S., Zhou Y. (2016). Effect of fiber content on the microstructure and mechanical properties of carbon fiber felt reinforced geopolymer composites. Ceram. Int..

[25] Lin T., Jia D., He P., Wang M., Liang D. (2008). Effects of fiber length on mechanical properties and fracture behavior of short carbon fiber reinforced geopolymer matrix composites. Mater. Sci. Eng. A.

[26] Yuan J., He P., Jia D., Yan S., Cai D., Xu L., Yang Z., Duan X., Wang S., Zhou Y. (2016). SiC fiber reinforced geopolymer composites, part 1: Short SiC fiber. Ceram. Int..

[27] Shin J.H., Kim D., Centea T., Nutt S.R. (2019). Thermoplastic prepreg with partially polymerized matrix: Material and process development for efficient part manufacturing. Compos. Part A Appl. Sci. Manuf..

[28] Hájková P. (2018). Kaolinite Claystone-Based Geopolymer Materials: Effect of Chemical Composition and Curing Conditions. Minerals.

